# A Cause of Facial Neuralgia

**Published:** 1873-06

**Authors:** 


### EDITORIAL, ETC.

---

*A Cause of Facial Neuralgia.*—Dr. Joseph H. Scales, of Newburn, Va., writes as follows:

"Enclosed you will find a tooth I extracted about three weeks ago for a lady. It is a *third lower molar*, and as you see, has five well defined roots, three of them ordinary size; though the posterior one, which entered the ramus of the jaw almost at right angles, was broken, leaving a portion of it. The person for whom I removed it, had been suffering the most excruciating agony from facial neuralgia for several months, and in the hands of a skilful physician, all the known remedies had been exhausted on her, without affording any relief. She was compelled to keep under the influence of opiates to get any rest at all. I determined to remove it, though the lady assured me that it had never ached, nor had it ever been even sensitive; while it is considerably decayed, the nerve is not exposed. The filling in the grinding surface was put in about three years ago, and it has never been any trouble since.

Since removing the tooth the neuralgia has left her entirely, and her general health is improving rapidly

